# The Bacterial ClpXP-ClpB Family Is Enriched with RNA-Binding Protein Complexes

**DOI:** 10.3390/cells11152370

**Published:** 2022-08-02

**Authors:** Georg Auburger, Jana Key, Suzana Gispert

**Affiliations:** Experimental Neurology, Medical Faculty, Goethe University, 60590 Frankfurt am Main, Germany; jana.key@kgu.de (J.K.); gispert-sanchez@em.uni-frankfurt.de (S.G.)

**Keywords:** mass spectrometry, substrate-trapping assay, BioID, POLDIP2, LRPPRC, GRSF1, PNPT1, MTIF2, ataxia

## Abstract

In the matrix of bacteria/mitochondria/chloroplasts, Lon acts as the degradation machine for soluble proteins. In stress periods, however, proteostasis and survival depend on the strongly conserved Clp/Hsp100 family. Currently, the targets of ATP-powered unfoldases/disaggregases ClpB and ClpX and of peptidase ClpP heptameric rings are still unclear. Trapping experiments and proteome profiling in multiple organisms triggered confusion, so we analyzed the consistency of ClpP-trap targets in bacteria. We also provide meta-analyses of protein interactions in humans, to elucidate where Clp family members are enriched. Furthermore, meta-analyses of mouse complexomics are provided. Genotype–phenotype correlations confirmed our concept. Trapping, proteome, and complexome data retrieved consistent coaccumulation of CLPXP with GFM1 and TUFM orthologs. CLPX shows broad interaction selectivity encompassing mitochondrial translation elongation, RNA granules, and nucleoids. CLPB preferentially attaches to mitochondrial RNA granules and translation initiation components; CLPP is enriched with them all and associates with release/recycling factors. Mutations in CLPP cause Perrault syndrome, with phenotypes similar to defects in mtDNA/mtRNA. Thus, we propose that CLPB and CLPXP are crucial to counteract misfolded insoluble protein assemblies that contain nucleotides. This insight is relevant to improve ClpP-modulating drugs that block bacterial growth and for the treatment of human infertility, deafness, and neurodegeneration.

## 1. Introduction

Proteolytic machines with AAA+ (ATPases associated with a variety of cellular activities) family members use energy from ATP to perform quality control of soluble proteins in all organisms and cell compartments, via refolding and subsequent degradation [1]. In the matrix of bacteria, mitochondria, and chloroplasts, the main activity to eliminate folded and misfolded soluble proteins seems to come from the AAA+ disaggregase and protease Lon [2], which can form a homo-hexameric ring structure. There is evidence that even the mitochondrial levels of the other major matrix AAA+ disaggregase, caseinolytic protease, chaperone component X (ClpX), may be determined by Lon [3,4]. Apparently, the abundance of soluble subunits within the respiratory chain and in the tricarboxylic cycle is determined by Lon levels in mouse embryonic fibroblasts (MEF) [5]. Lon is associated also with the mtDNA/TFAM protein complex, being necessary to maintain the mitochondrial nucleoid [6]. Its multiple roles make its mammalian ortholog LONP1 essential for life (mammalian proteins will be written in uppercase letters (e.g., LONP1), human genes in uppercase with italics (e.g., *LONP1*), rodent genes with lowercase (e.g., *Lonp1*), while lowercase letters are always used for bacterial proteins (e.g., Lon), bacterial genes (e.g., *lon*) and gene deletions (Δ*lon*)). In mice, the homozygous *Lonp1* deletion leads to early embryonic lethality [7].

In contrast, the homozygous deletion of CLPP as the only other mitochondrial matrix peptidase triggers only a smaller organism size, e.g., in mice [8], with a combination of complete infertility and age-associated deafness in autosomal recessive inheritance (Perrault syndrome or PRLTS in human nomenclature). However, CLPP-depletion can extend healthy lifespan, e.g., in the eukaryotic microorganism *Podospora anserina* [9]. The homozygous CLPP deletion in mouse embryonic fibroblasts (MEF) triggers proteome accumulations mainly for mitoribosomal subunits [5].

Many efforts have been made to understand in detail what functions are performed by ClpX and ClpP and by the AAA+ disaggregase ClpB as a minor pathway member, which cannot be executed by Lon. These functions have to be crucial, to explain why these factors with caseinolytic activity were conserved throughout phylogenesis from bacteria to mitochondria and chloroplasts. Clearly, Clp family roles become prominent only in stress periods such as heat shock [10] and are thought to compensate for the toxic effects of insoluble protein aggregates [11]. It was shown that the recognition/selective binding/refolding by ClpB and ClpX—or alternatively, linearization/translocation into the degradation chamber by ClpX—is performed by the AAA+ disaggregase activity, while their cleavage to small peptides is carried out by ClpP homo-heptameric rings [1]. ClpB is not stably associated with ClpP, but its unfolding activity will prevent the accumulation of targets in insoluble inclusion bodies, and thus enhance their proteolysis by any matrix protease, as argued previously [11]. In contrast, ClpX is stably associated and acts as ATPase to provide the chemical energy needed for ClpP-driven proteolysis. This interaction occurs within a barrel-like complex of two adjacent homo-heptameric ClpP rings surrounded on either side by homo-hexameric ClpX rings [12].

The protein targets of this ClpXP proteolytic machine and of the non-associated disaggregase ClpB remain unclear; a strong data variance between organisms has led to various disputes. A recent review on ClpP’s roles focused on eukaryotic data and summarized in its first figure [13] that only 27 putative CLPP degradation substrate proteins are consistently accumulated both in humans and mice (ALDH6A1, NFS1, MUT, ACADSB, CBR4, NDUFA4, SDHA, SDHC, COX5A, COX5B, COX6A1, NDUFAF1, CS, ACO2, IDH3B, ACSS1, CARS2, PET112/GATB, NARS2, QRSL1, WARS2, TFAM, SSBP1, POLDIP2, POLG2, CLPX, ALAS1), with a strong preponderance of respiratory chain proteins. Among them all, only ALDH6A1, NFS1, ACADSB, CS, and ACO2 are consistent also with data from the eukaryotic microorganism *Podospora anserina* [13]. All of them are soluble proteins in the mitochondrial matrix; all have metabolic functions, suggesting that CLPP acts in stress periods to adapt the nutrient situation of cells. However, these factors have no putative interaction subdomains or associated cofactors in common. Furthermore, the phenotypes triggered by mutations in these factors have no overlap with CLPP depletion phenotypes. In patients, CLPP mutations cause complete infertility due to primary ovarian insufficiency and non-obstructive testicular azoospermia, together with early sensorineural progressive deafness, sometimes followed by ataxia and leukodystrophy, inherited in an autosomal dominant manner, a condition known as Perrault syndrome type 3 (PRLTS3) [14,15]. Perrault syndrome is genetically heterogeneous, but most disease proteins have prominent roles in mitoribosomal translation/RNA processing [16]. In contrast, quite different phenotypes are triggered by mutations in ALDH6A1 (developmental delay due to methylmalonate semialdehyde dehydrogenase deficiency, with dysmorphic facial features and hepatoencephalopathy), NFS1 (combined oxidative phosphorylation deficiency 52 with cardiorespiratory and liver failure), ACADSB (developmental delay due to 2-methylbutyryl-coA dehydrogenase deficiency, with vomiting, breathing difficulties, seizures), CS (critical illness polyneuropathy), ACO2 (optic atrophy type 9, or infantile cerebellar-retinal degeneration) (see respective entries in the GeneCards database (https://www.genecards.org/; last accessed on 5 July 2022). After comparison with data from the bacteria *Escherichia coli* and *Bacillus subtilis*, no consistent CLPP substrate candidate seems to remain, despite the maximally conserved sequence and structure of CLPXP across phylogenesis. It is difficult to reconcile the metabolic concept with the specific and crucial role of ClpXP for bacterial virulence and alpha-hemolysin secretion, which makes ClpXP a prime target for anti-bacterial drug design [17,18,19]. Methodological problems behind this controversy firstly include the assumption that accumulated factors in global proteome profiles represent degradation substrates, without testing if they are due to transcriptional induction or to indirect effects, and secondly, the contamination of substrate-trap assays with very abundant proteins.

The present study aims (1 + 2) to review each report of putative ClpXP-ClpB substrates, separating different techniques (trap assays versus global proteome profiles) and organisms, (3) to add insights from high-confidence knowledge on human CLPXP-CLPB interactomes, by meta-analyses of statistical pathway enrichments, (4) to exploit novel mouse protein complexomics studies, by meta-analyses of protein abundance and migration patterns in published data, and (5) to compare the findings with recent genotype-phenotype correlations. It is important to consider the advantages and limitations of each approach:

(1) The trapping approach takes advantage of observations in *E. coli* that inactivated ClpP stays associated with substrates that were selected by ClpX and translocated into the degradation chamber [20], but of course, very abundant soluble matrix proteins will also be observed in subsequent mass spectrometry as contaminants. Given that organisms are adapted to different environments and therefore have different expression profiles, the artifacts should vary upon analysis of many species, while true ClpP degradation substrates should be detected with high consistency.
(2) Global proteome profiling represents a less laborious approach that can quantify steady-state accumulations also in complex tissues. However, it remains unclear if any protein accumulation is due to transcriptional or translational activation or to retarded protein turnover due to other reasons, so many effects may be indirect and may not represent true CLPP targets.
(3) Protein–protein interaction data may be useful, given that the association of a misfolded target protein with an unfoldase/disaggregase or with a peptidase during the degradation process would be more stable than the “kiss-and-run” interaction of targets with their protein kinases. Therefore, it may be possible to identify such associations by biochemical high-confidence interaction studies. As an abundant source of observations on CLPB/CLPX/CLPP interactomes, we now decided to employ the *Homo sapiens* mitochondrial proximity interaction network detected by the BioID technique (Table S4 in [21]) and evaluate statistically/visually in a meta-analysis any enrichments, using the STRING webserver (https://string-db.org/ (accessed on 20 July 2022)) [22]. This effort complements our earlier work, where we had taken the complete BioGRID dataset into account [15]. However, the use of tagged and overexpressed recombinant proteins may trigger artificial interactions that occur in non-physiological sub-compartments of the cell.
(4) The study of the assembly of endogenous mitochondrial proteins into complexes was optimized recently via blue-native (BN) or high-resolution clear native (CNE) gel electrophoresis in two dimensions or one dimension, followed by mass-spectrometry identification of the components [23]. The analysis of comigration patterns has greatly helped to define assembly intermediates of the respiratory chain [24], and mitochondrial data generated by such efforts may help to clarify the associations of CLPB and CLPXP. The advantage of this approach is the ability to observe endogenous proteins in their physiological association and their altered assembly/migration within protein complexes upon mutation impact. Their limitation consists of only quite abundant proteins and stable interactions being detectable.
(5) The notion that CLPXP targets the mitochondrial RNA processing protein complexes is indeed supported by genotype–phenotype correlation data. However, any phenotype overlap is not necessarily triggered by protein–protein interactions that are in common.

Altogether, all five lines of evidence converge to indicate that a strong association with specific ribonucleoproteins (RNPs) seems to characterize the CLPXP-CLPB proteins.

## 2. Substrate-Trapping Assays

In three different bacteria, trap assays were quite successful and generated consistent results, but only a small part of the findings were reproduced in eukaryotes (see Table 1 below). One illustrative example is the ClpP dependence of the DNA repair factor RecA within the SOS response program to mutagenic stress conditions [25] in bacteria, which cannot be reproduced in eukaryotes given that the two mitochondrial membranes and the two nuclear membranes separate mitochondrial CLPP from the RecA homolog DMC1. However, the CLPP-dependent SOS program regulates also translation elongation/recycling factor PrfB/PrfC expression [26], a stress response that might be conserved in the mitochondrial matrix.

*E. coli* and other bacteria have long been the workhorse for the understanding of the Clp family and for the achievement of structural insights. The first unbiased screen via substrate-trapping assays, to identify the degradation targets of ClpP, was reported for *E. coli* by the team of Tania Baker in 2003 [27]. More than 50 proteins were trapped in the presence of ClpX and the absence of ClpA, including transcription factors, metabolic enzymes, as well as starvation and oxidative stress response elements. It is relevant to note here that these enrichments included the disaggregase ClpX, the RNA polymerase RpoS (sigma factor, which controls the bacterial expression of hundreds of stress response components), several other transcription factors, as well as the translation elongation factor TufB and GTP-binding factors such as the cell division regulator FtsZ [27]. A subsequent evaluation of ClpXP function in *E. coli* showed it to prevent the accumulation of FtsZ aggregates in vivo under extreme thermal stress [28].

As a next step to characterize ClpP-dependent degradation substrates in *E. coli*, the team chose DNA damage as a stress condition, to then document the trap assay proteome in more depth [25]. Prominent accumulations were detected for the disaggregase ClpX again, and attention then focused on the nucleotide excision repair subunit UvrA and the DNA double-strand break repair factor RecN, both of which are part of the SOS response program. However, it should be also noted that LepA and FusA as interactors of the translation elongation factors TsfB/TufB, as well as the translation initiator InfB accumulated more than 2-fold, together with several ribosomal subunits (RpsA, RpsG, RplD, RplF, RplJ, RplO) and transcription modulators (DeaD, Fnr, Pnp, RapA, Rho, RpoA, RpoD) [25].

In *Caulobacter crescentus*, a substrate-trap study of ClpP-deleted bacteria found enrichments for 127 proteins, among which energy production, cell wall, translation, post-translational control, and transcription were particularly frequent pathways. Apart from ClpX/ClpA, co-purification was confirmed for several DNA-binding factors (e.g., DnaJ, GyrB, and RecA), but also observed for the translation initiation factor InfB and the GTP-binding cell division regulator FtsZ again [29]. Important findings are compiled in Table 1, where putative ClpP substrates that were identified consistently in at least two trap assays in different organisms are shown, together with supporting data from global protein profiles from additional species.

In *Staphylococcus aureus*, a substrate-trap study identified numerous candidate targets of ClpP-mediated degradation. They were grouped in the functional categories energy metabolism, biosynthesis of cofactors/prosthetic groups/carriers, DNA metabolism/repair (e.g., DnaJ, GyrB, RecA, and UvrA), and protein synthesis/fate. Here, it is noteworthy that they included (see Table 1) several transcriptional regulators (e.g., the GTP-sensing transcriptional pleiotropic repressor CodY, the RNA polymerase RpoB, the transcription termination factor Rho, the mRNA degradation enzyme PnP), the aspartyl/glutamyl-tRNA amidotransferase subunits GatA and GatB, again the translation elongation factor TsF, and the GTP-binding cell division regulator FtsZ [30]. A previous mechanistic and functional study had already shown that the absence of ClpX enhanced misfolding, but extended survival and that ClpP and ClpX mutation in *S. aureus* attenuated virulence in a murine skin abscess model, in association with reduced transcript levels of major secreted proteins [31].

In the eukaryotic fungus *P. anserina*, a substrate-trap and differential proteomics analysis of ClpP-ablated microorganisms documented co-purification with the ClpP-Trap-tag and the ClpP-WT-tag for 47 putative substrates. They included multiple enzymes of amino acid/fatty acid metabolism, the pyruvate dehydrogenase complex, and the tricarboxylic acid cycle, as well as the electron transfer complex I. Apart from these pathways of energy metabolism, the homolog of translation elongation factor TUFM also showed stronger enrichment with the Trap-tag than with the WT-tag, while the homolog of mtRNA methylation factor SHMT2 was associated with both (see Table 1). Among the 20 proteins that co-purified exclusively with the Trap-tag were the disaggregase ClpX, with the homologs of ribonucleoprotein HNRNPA2B1, mitochondrial translation elongation factor GFM1, and mitoribosomal subunit YMR31 [32].

The scarcest insights came from a CLPP-substrate trapping study of *Homo sapiens* AML cancer cells. They were observed to exhibit reduced growth and viability upon CLPP depletion, potentially enhancing patient survival [33]. For further mechanistic insights, the authors overexpressed wildtype and inactivated CLPP with a BioID proximity-labelling-tag compared to another mitochondrial control protein, to trap CLPP-interacting proteins and identify them by mass spectrometry. The interaction of 49 mitochondrial proteins with CLPP was documented in this manner, with enrichment of metabolic pathways and oxidative phosphorylation. Our meta-analysis with the STRING webserver revealed an enrichment also for nucleotide binding (q = 0.007), including 22 proteins such as CLPX together with 4 other components of the mitochondrial nucleoid (TFAM, SSBP1, POLDIP2, and POLG2), 5 components of tRNA aminoacylation (CARS2, NARS2, WARS2, QRSL1, and GATB), as well as 2 translation release/recycling factors (MRRF and MTRF1). Unfortunately, the consistency of bacterial findings with human findings by this variant approach was unexpectedly low (see Table 1).

## 3. Proteome Profiles

A wealth of global proteome profiles has been generated over the past year, providing rapid survey data on putative ClpP substrates in various organisms with very different metabolism, but with the caveat that mRNA inductions are important confounders.

Indeed, in *B. subtilis*, efforts to identify ClpP degradation substrates (via 2D-polyacrylamide gel electrophoresis and MALDI-TOF peptide mass fingerprinting) concluded that most protein accumulations were due to excess transcriptional activity downstream from the stress response factors sigma-B and Spx. An additional pulse-chase radiolabeling approach to confirm absent protein decay identified 16 candidate ClpP degradation substrates, including four amino acid tRNA synthases (e.g., MetS and ProS), and several enzymes in the metabolism of nucleotides, amino acids, and carbohydrates [34]. A follow-up study from the same team with an 8 h time course identified about 80 putative ClpP substrates, and this effort yielded excellent comparability with substrate-trap assay data (Table 1). Proteins with >1.5-fold elevation of degradation stability, in ΔClpP and ΔClpX cells entering the stationary phase due to glucose restriction, were mainly involved in the metabolism of nucleotides and amino acids. It is relevant here to stress that they included the ribosomal translation elongation factors TufA (ortholog of human TUFM) and Efp (a translation elongation factor that binds to paused ribosomes requiring assistance with the formation of oligo-prolines [35]), as well as the RNA polymerase subunit RpoB [36].

Simple proteome profiling of *Staphylococcus aureus* bacteria in high-iron growth conditions versus low-iron growth-arrest conditions, using two-dimensional difference gel electrophoresis (2D-DIGE) and mass spectrometry, documented ClpP-dependent changes in several stress-response pathways, general metabolism, and also electron transport [37]. It should be noted that ΔClpP cells showed an increased abundance of FusA (>6-fold, ortholog of GTP-associated ribosomal translocation factor GFM1 in mammals) and of several transcription factors such as the GTP-sensing growth regulator CodY (>3-fold) during growth. In contrast, growth-arrest selectively triggered the accumulation of disaggregase ClpB (between 3- and 26-fold), together with the translation elongation factor TuF (4.5-fold), the protein and nucleotide Maillard adduct deglycase HacH (4.5-fold), and the protein membrane translocase SecA (4.3-fold) [37].

In the chloroplast of the model plant organism *Arabidopsis thaliana*, proteome profile alterations upon depletion of Clp protease were studied with 2D-DIGE/mass spectrometry and with a protein degradation assay with a time course over only 3 h, after heat shock stress [38]. This approach identified 19 candidate substrates, with enrichment in metabolic pathways, RNA maturation, protein synthesis, as well as recycling processes. They included the translation elongation factors EF-G (ortholog of bacterial FusA and mammalian GFM1)/EF-Tu (ortholog of bacterial TufB and mammalian TUFM), the DEAD-box ATP-dependent RNA helicase 3 (emb1138), as well as RNA-binding proteins At2g37220 and CP29.

In *Mus musculus*, animals with deficiency of CLPP were characterized recently, and in *Homo sapiens*, the investigation of patients with Perrault syndrome revealed underlying CLPP mutations. Both developments made the analysis of mammalian cell types possible. The first CLPP-null global proteome profile was reported for mouse heart mitochondria and convincingly demonstrated an accumulation of mitoribosomal subunits [39]. Both the rRNA chaperone ERAL1 and the translation elongation factor GFM1 were accumulated with a high effect size and significance, while a downregulation of TUFM and an upregulation of TSFM were minor, failing to reach significance. Both ERAL1 and GFM1 were not induced transcriptionally, and their protein turnover rates upon cycloheximide exposure were lost after CLPP deletion. ERAL1 was claimed to be more important than GFM1 for pathology, in view of its abnormally strong and dispersed association with mitoribosomes and the observation of a homozygous missense mutation of ERAL1 in three PRLTS patients [39,40]. Accumulation was also observed for PNPT1 and MRPP1, two components of the RNAse P complex, which is responsible for mitochondrial RNA import and for clearance of oxidized RNA. Mutations in this complex cause infertility and deafness.

Somewhat more sophisticated proteome profiles (without transcriptional studies) of CLPP-null mouse heart mitochondria via N-terminal peptide analysis and cycloheximide chase over 10 h identified only the CLPX-stabilizing factor POLDIP2 [4] and the mitoribosomal proteins MRPS22/MRPL13 and MRPL18 as putative CLPXP substrates, together with matrix enzyme OAT, chaperone HSPA9, and respiratory chain subunit UQCRC1 [41].

When human CLPP-mutant patient skin fibroblasts were assessed and the proteome profiles were assessed for consistency with mouse CLPP-null fibroblasts (which derive most energy from glycolysis and proliferate steadily) and mouse CLPP-null brain (most energy from respiration; the brain contains many post-mitotic neurons), the changes in label-free proteome profiles upon CLPP mutation were analyzed in depth to define consistent CLPP-degradation targets [15], but without time course studies or transcriptome analyses. These experiments already took into account what information for CLPX–protein interaction is available in the BioGrid database. The study concluded that the ribonucleoproteins GFM1, GRSF1, LRPPRC, and POLDIP2 show the highest reproducibility and accumulation ratios, while ERAL1 accumulation was not consistent [15].

## 4. Protein Interaction Data

Proteins with low abundance will not easily be detected by both previous approaches, but can be represented well in protein interaction experiments where they are overexpressed as baits.

In human cancer cell lines, efforts to identify CLPP degradation substrates were carried out, using a mitochondrial-matrix-targeted construct for APEX-mediated proximity biotinylation of proteins there, combined with acute knockdown over 48 h of CLPP versus LONP1. Subsequent proteome mass spectrometry analyses demonstrated the accumulation of 82 CLPP candidate targets within the pathways of oxidative phosphorylation, the TCA cycle, as well as amino acid, and lipid metabolism [42]. Here, it is relevant to note that potential CLPP targets included TFAM, SSBP1, MRPS7, MRPL13, and IARS2. However, the authors did not assess whether transcriptional inductions or secondary effects were responsible for these accumulations, and they failed to study the conservation of effects across phylogenesis. The study then focused on the pyridoxal-phosphate-dependent enzyme SHTM2, which can be degraded by LOPN1 or CLPXP according to their findings [42].

In the BioID dataset generated in modified human HEK293 cells, we did not trust the tagged overexpression data where excess bait may mislocalize extra-mitochondrially, but preferred to include only associations where the endogenous protein of interest was identified as prey. This approach showed CLPB, CLPX, and CLPP to have shared interactions, mostly with proteins in the mitochondrial RNA processing pathway. At the same time, some differences existed and may be considered to point at specific roles for each prey.

**Figure 1:**
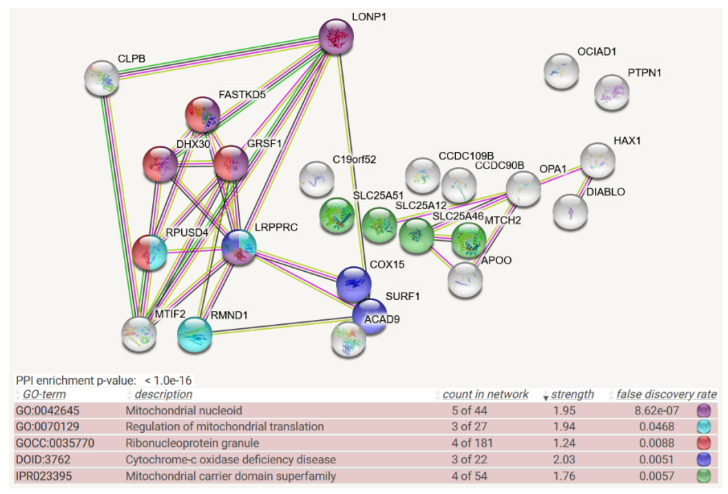
**Mitochondrial BioID network filtered for interactions with CLPB as prey, shown as STRING diagram**. Schematic visualization of each protein–protein interaction (PPI) component as bullet, the enriched pathways reflected by bullet coloring as detailed in the lines below. (The text columns represent pathway code, pathway description, network count, enrichment strength, false discovery rate statistics on enrichment significance, and color code. The single test of PPI enrichment is given as the *p*-value, while the multiple testing of pathway enrichments below is evaluated by false discovery rate q-values.) The association evidence strength is highlighted as colored lines. CLPB and LONP1 as mitochondrial matrix AAA+ unfoldases are shown in the upper left. Interaction between CLPB and MTIF2 is well documented in other STRING organisms (anti-tag coimmunoprecipitation in human and bacteria (see [43,44,45,46,47]), coexpression and genomic neighbors in other organism). FASTKD2, GRSF1, DHX30, and RPUSD4 are already classified as components of ribonucleoproteins; recent experiments showed also LRPPRC to associate with mitochondrial RNA granule factors [48]. LRPPRC, RPUSD4, and RMND1 are modulators of mitoribosomal translation. The respiratory chain components COX15, SURF1, and ACAD9 may reflect the fact that translation efficiency and fidelity are needed particularly for the cytochrome-c oxidase complex IV, as the rate-limiting step of oxidative phosphorylation. Apart from the enrichment of the RNA processing pathway, mitochondrial membrane carriers including the NAD+ carrier SLC25A51, the S-adenosylmethionine carrier SLC25A46, calcium-associated carriers SLC25A12 and MTCH2, and putative calcium uniporters CCDC90B/CCDC109B were also observed in unexpected numbers. This may reflect a submitochondrial position of CLPB near the inner membrane.

For CLPB filtered as prey of BioID-tagged recombinant baits, the STRING meta-analysis generated a highly significant (q < 1.0 × 10^−16^) enrichment for protein–protein interactions, and almost all 24 baits are normally localized to mitochondria. Prominent pathways and gene ontology (GO) terms showed a cluster of interactions at the mitochondrial RNA granule (Figure 1). For CLPB as overexpressed tagged bait, in contrast, the analogous analysis defined 775 candidate interactors (the resulting STRING figure would show too many bullets, which make interaction lines and protein symbols invisible, making it useless to be included even as a Supplement), among which 656 proteins localize to the cytoplasm and 67 to chromatin. Interestingly, also in this “bait-condition”, the prominent pathways and GO terms listed mRNA binding (q = 5 × 10^−173^), ribonucleoprotein complex (q = 8 × 10^−106^), spliceosome (q = 1 × 10^−31^), ribosome (q = 5 × 10^−46^), and translation initiation (q = 6 × 10^−57^). Thus, prey endogenous CLPB in mitochondria associates preferentially with BioID-tagged baits that form part of ribonucleoprotein complexes, and even upon overexpression of BioID-tagged CLPB as bait in the cytoplasm and nucleus, this preferential interaction with ribonucleoprotein complexes is maintained.

**Figure 2:**
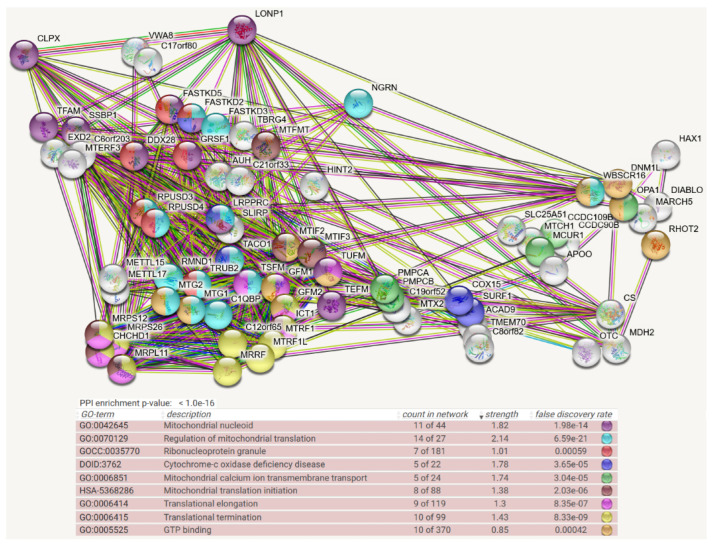
**Mitochondrial BioID network filtered for interactions with CLPX as prey, shown as STRING diagram**. Schematic visualization of each protein–protein interaction (PPI) component as bullets; the enriched pathways reflected by bullet coloring as detailed in the lines below. (Text columns represent pathway code, pathway description, network count, enrichment strength, false discovery rate statistics on enrichment significance, and color code as before. The single test of PPI enrichment is given as the *p*-value, while the multiple testing of pathway enrichments below is evaluated by false discovery rate q-values.) The association evidence strength is highlighted as colored lines. CLPX, LONP1, and VWA8 as mitochondrial matrix AAA+ unfoldases are shown in the upper left. CLPX interactions are well documented already with TFAM (evidence in reference [49], as well as coexpression in other organisms) and SSBP1 (evidence: anti-tag coimmunoprecipitation, coexpression in other organisms) as mtDNA-associated factors, with GFM1 (anti-tag coimmunoprecipitation, coexpression in other organisms), TUFM (anti-tag coimmunoprecipitation, genomic neighbors in other organisms), MTIF2 (anti-tag coimmunoprecipitation, coexpression, and genomic neighbors in other organisms), LRPPRC (anti-tag coimmunoprecipitation, coexpression in other organisms), MTG2 (coexpression and genomic neighbors in other organisms), and ICT1 (anti-tag coimmunoprecipitation, coexpression in other organisms) as mtRNA-associated factors, as well as PMPCA/PMPCB (anti-tag coimmunoprecipitation and coexpression in other organisms) as mitochondrial protein import processing factors and LONP1 as a bulk protein degradation factor. CLPX is more abundant than CLPB, and more bait interactors were identified for it. Most interactors of CLPB were found also to interact with CLPX, but only CLPX showed novel pathway enrichments with factors of mitochondrial transcription (e.g., TFAM, SSBP1, MTERF3, TEFM, DDX28), RNA processing/degradation (GRSF1, DDX28, FASTKD5, FASTKD2, FASTKD3, TBRG4, XED2), RNA methylation/formylation (METTL15/17, MTFMT), translational elongation/termination (TSFM, GFM2, MTRF1, MTRF1L, MRRF, C12orf65, RPUSD3, RPUSD4, RMND1, TRUB2, MTG1, C1QBP, TACO1, LRPPRC, SLIRP, FASTKD3, FASTKD2), ribosomal subunits (MRPS12, MRPS26, CHCHD1, MRPL11), GTP-binding (e.g., MTIF2, MTG2, GFM1, TUFM, DNM1L, OPA1), fusion/fission (e.g., OPA1), and matrix enzymes (OTC; MDH2; CS). The interaction with AUH is curious, since mutations in this enzyme cause 3-methylglutaconic aciduria, which was described as a feature of CLPB-mutant patients [50,51,52].

For CLPX as prey also, the STRING meta-analysis generated a highly significant (q < 1.0 × 10^−16^) enrichment for protein–protein interactions, and almost all 73 baits are normally localized to mitochondria. Prominent pathways and Gene Ontology (GO) terms showed clusters of interactions ranging from the nucleoid via mitochondrial RNA granules to mitoribosomal translation (now including the initiation, elongation, and termination steps) and extending to fusion/fission machinery and matrix enzymes. Interestingly, the association of proteins with GTP was enriched (Figure 2). CLPX was not employed as bait in the mitochondrial BioID survey [21].

**Figure 3:**
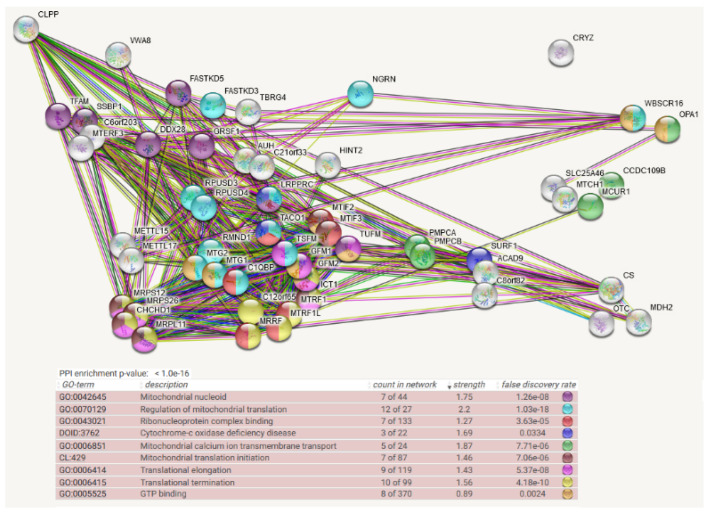
**Mitochondrial BioID network filtered for interactions with CLPP as prey, shown as STRING diagram**. Schematic visualization of each protein–protein interaction (PPI) component as bullets; the enriched pathways reflected by bullet coloring as detailed in the lines below. (Text columns represent pathway code, pathway description, network count, enrichment strength, false discovery rate statistics on enrichment significance, and color code as before. The single test of PPI enrichment is given as *p*-value, while the multiple testing of pathway enrichments below is evaluated by false discovery rate q-values.) The association evidence strength is highlighted as colored lines. Although CLPP is more abundant than CLPX, fewer bait interactors were identified for it, in agreement with the notion that target engagement und unfolding by CLPX is a lengthy process, and only for some targets, it is a precondition for subsequent quick degradation by CLPP. Almost all candidate interactors were part of the CLPX network. ClpP as peptidase is shown in the upper left, together with the mitochondrial matrix AAA+ unfoldase VWA8, while CLPX was not included as bait in this survey. Again, CLPP interactions are well documented already with TFAM (coexpression in other organisms) and SSBP1 (anti-tag coimmunoprecipitation, coexpression in other organisms) as mtDNA-associated factors, LRPPRC (anti-tag coimmunoprecipitation), MTG2 (anti-tag coimmunoprecipitation, coexpression and genomic neighbors in other organisms), MTIF2 (anti-tag coimmunoprecipitation, coexpression and genomic neighbors in other organisms), TUFM (anti-tag coimmunoprecipitation, coexpression and genomic neighbors in other organisms), and ICT1 (coexpression and genomic neighbors in other organisms) as mtRNA-associated factors, as well as PMPCA/PMPCB (anti-tag coimmunoprecipitation and coexpression in other organisms) as mitochondrial protein import processing factors, and SURF1/CS/OTC (coexpression in other organisms) as mitochondrial matrix soluble proteins. CLPP interactions previously documented in other species that are not conserved for CLPX include MRPS12 (anti-tag coimmunoprecipitation, coexpression in other organisms), MRRF (coexpression, genomic neighbors in other organisms), C12orf65 (coexpression, genomic neighbors in other organisms), RMND1 (coexpression in other organisms), and TACO1 (coexpression, genomic neighbors, and genomic co-occurrence in other organisms).

For CLPP as prey also, the STRING meta-analysis generated a highly significant (q < 1.0 × 10^−16^) enrichment for protein–protein interactions, and almost all 54 baits normally localize to mitochondria. Prominent pathways and Gene Ontology (GO) terms showed clusters of interactions again ranging from the nucleoid via mitochondrial RNA granules to mitoribosomal translation (again including initiation, elongation, and termination steps) and extending to fusion/fission machinery and matrix enzymes (Figure 3). Strong preexisting evidence in additional species for CLPP-selective interactions with translation fidelity/release/recycling/surveillance/efficiency control factors such as MRPS12 (controls decoding fidelity and susceptibility to aminoglycoside antibiotics)/MRRF (releases ribosomes from mRNA at termination)/C12orf65 (=MTRFR, ejects unfinished nascent chains and peptidyl transfer RNAs from stalled ribosomes)/RMND1 (ribosome surveillance/maintenance factor)/TACO1 (translational activator of COX1) (see Figure 3 legend). This might point to incomplete translation products such as COX1 fragments that resist CLPX-mediated refolding and then associate with the above factors before they are subjected to CLPP-mediated degradation. CLPP was not employed as bait in the mitochondrial BioID survey [21].

## 5. Complexomics Data

The detection of endogenous proteins within complexes and of migration anomalies triggered for several proteins in parallel by the same mutation is an enormous advantage of complexome studies, but again, the detection is limited to abundant factors.

Colored interaction lines in the CLPB-interactome STRING diagram (Figure 1) highlight a strongly conserved, known association of CLPB with MTIF2 family members. This was first observed in *E. coli* complexomics profiling efforts. In this study employing 2D blue native gels, ClpB comigrated with the MTIF2 ortholog IF-2 (translation initiation factor), encoded by the *infB* gene, which protects N-formylmethionyl-tRNA and promotes its binding to the 30S pre-initiation complex, where this tRNA binds to the start codon, as well as with the 30S small ribosomal subunit S1 (encoded by the *rpsA* gene, unfolding structured mRNAs to associate initiation codon with ribosome), and DnaK (molecular chaperone orthologous to HSPA9) [44]. Furthermore, the interaction lines in Figure 1 reflect CLPB and MTIF2 homologs to show co-expression in several organisms and to have neighboring genomic positions in several organisms.

In *Mus musculus*, mitochondrial complexomics profiles in wildtype and CLPP-deleted hearts were recently documented [53]. We performed a meta-analysis of proteins of interest, regarding abundance across all gel slices and their overlap and migration range. The data revealed accumulation of CLPB (2-fold) and CLPX (6-fold) in mutant tissue. The migration positions of CLPB appeared unchanged by the CLPP deficiency, and similarly, accumulations of EF-Ts (encoded by TSFM, 1.5-fold), AUH (1.5-fold), HINT2 (1.8-fold), CHCHD1 (3-fold), MRPL11 (1.5-fold), COX15 (1.5-fold), and MARCH5 (2-fold) were observed with unchanged distribution in the gel. In comparison, accumulated CLPX in the mutant acquired a disperse migration across many low-molecular-weight slices, and this pattern modification was observed also for the CLPX stabilizer POLDIP2 (accumulated 14-fold), GRSF1 (15-fold), GFM1 (8-fold), TBRG4 (=FASTKD4, 5-fold), LRPPRC (4-fold), GFM2 (3-fold), VWA8 (2-fold), and MRPS26 (accumulated in mutant, versus a value of 0 in WT control, yielding an infinite fold-change). TFAM, SSBP1, FASTKD2/3/5, SLIRP, TUFM, TACO1, C1QBP, PMPCA/B, MTX2, SURF1, ACAD9, the mitochondrial membrane carriers, and matrix enzymes MDH2/CS were not accumulated. EXD2, MTERF3, MTIF2, DHX30, FASTKD2, MTFMT, RPUSD3/4, METTL15/17, MTIF3, TEFM, RMND1, TRUB2, MTG1/2, ICT1, MTRF1/1L, MRRF, MRPS12, NGRN, RMND1, RPSA, TMEM70, and OTC were not detected.

While the above-mentioned global proteome profiles of mammalian CLPP-null tissues had not provided clear insights into the role of CLPB and CLPXP for RNPs, the mouse complexomics findings thus provide strong and valuable support for this concept. Therefore, it is important to ask if human observations are also in agreement, and therefore, we turned to the analysis of mutation effects in patients.

## 6. Genotype–Phenotype Correlation

Below, we discuss evidence that mitochondrial causes of infertility due to meiotic arrest are mostly triggered by mtDNA mutations/pathology, while mitochondrial causes of sensorineural deafness are usually caused by mtRNA mutations/pathology.

CLPB mutations can lead to relevant phenotypes already at birth, while weaker mutations can go almost unnoticed [54,55]. This is noteworthy, since CLPB shows low protein levels in the human organism (mean abundance according to a “genotype–tissue-expression” database; see https://gtexportal.org/home/; last accessed on 4 July 2022, is about 10 TPM). In such patients, age-associated brain white matter degeneration (leukodystrophy upon brain imaging, with prominent ataxia upon neurological examination) is present, fetal or congenital microcephaly can appear, cataracts develop, and blood neutropenia can be severe or intermittent [50,56,57,58]. Even in heterozygous carriers, symptoms may appear, with neutropenia often constituting the only anomaly [59]. In the catalog of Genome-Wide Association Studies (GWAS) (https://www.ebi.ac.uk/gwas/genes/; last accessed on 4 July 2022), where mild effects of gene variants are expected to appear, tentative effects of CLPB on body height, obesity, diastolic blood pressure, heart rate response to exercise, electrocardiogram features, blood platelets, and adolescent idiopathic scoliosis were reported. Mutations of MTIF2, FASTKD5, and RMND1 in patients have not yet been reported, but MTIF2 was implicated in conduct disorders and in aminoglycoside-induced deafness (see GeneCards database, https://www.genecards.org/; last accessed on 5 July 2022). Mutations in LRPPRC lead to a respiratory complex IV (COX-I) deficiency syndrome named MC4DN5, with adult ataxia [60]. DHX30 mutations lead to NEDMIAL with ataxia and psychomotor retardation [61]. TSFM mutations cause COXPD3 with complex IV deficiency and subsequent ataxia [62]. COX15 mutations result in MC4DN6 with complex IV deficiency and Leigh syndrome leukodystrophy [63]. HSPA9 and RPUSD4 were implicated in sideroblastic anemia according to the GeneCards database. Thus, mutations in the putative CLPB interactome frequently cause neurodegenerative processes with clinical ataxia, rarely deafness, and sometimes, different blood anomalies.

CLPX has intermediate tissue levels in humans (mean abundance 20 TPM). Only one mutation has been described so far [64], which impairs its CLPP-independent protein–protein interaction with the heme biosynthesis enzyme 5-aminolevulinic acid synthase (ALAS) [65], causing erythropoietic protoporphyria 2 (leading to acute skin photosensitivity, mild microcytic anemia, and rarely, severe liver disease). Furthermore, in the GWAS catalog, only an impact of CLPX on red blood cells and hemoglobin is documented. So far, there is no genetic evidence on CLPX mutations affecting CLPP degradation substrates, so the patient data are clearly incomplete, and further CLPX variants will presumably be reported in the future either in congenital mitochondriopathies or as modifiers of varying phenotypes. For the CLPX target candidate SSBP1, mutations were identified in patients with sensorineural deafness [66]. For GFM1, mutations cause COXPD1. Biallelic variants in a child caused hearing impairment, documented as vestibule-cochlear affection upon brainstem auditory-evoked potential studies [67]. In the GWAS catalog, GFM1 appears associated with the electrocardiographic PR interval, diastolic blood pressure, and diarrhea. Mutations in GFM2 result in COXPD39 with complex IV deficiency and subsequent ataxic hypotonia followed by Leigh syndrome leukodystrophy [68]. For TUFM, mutations trigger COXPD4 with complex IV deficiency and rapidly progressive encephalopathy [69]. MTG2 is associated with DFNA67, an age-associated deafness (see GeneCards entry). For the CLPX target candidate GRSF1, a heterozygous gene variant was reported in a case with developmental regression, intellectual disability, and refractory epilepsy [70], while the GWAS catalog only reports an association with red blood cell distribution width. For the CLPX target candidate LRPPRC, it is interesting to note that biallelic variants were observed in a patient with infertility due to premature ovarian insufficiency [71]. In the GWAS catalog, many associations were observed for LRPPRC variants, mainly with total cholesterol, alcohol consumption, antisocial behavior, intelligence, and grip strength. Finally, for the CLPX target candidate POLDIP2, no gene variants in patients have been documented so far. The GWAS catalog for POLDIP2 reports only a correlation with blood protein levels. Thus, mutations in the putative CLPX interactome again lead to neurodegenerative processes with high frequency, rarely deafness, but also to anemia (presumably via ALAS) and even ovarian failure.

**Figure 4:**
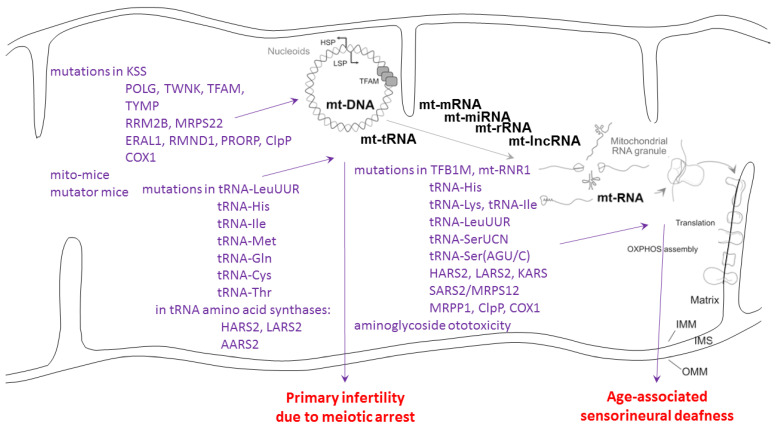
**Genotype–phenotype data for mitochondrial causes of infertility and deafness.** HSP: heavy strand promoter; LSP: light strand promoter; TFAM: transcription factor A in mitochondria; IMM: inner mitochondrial membrane; OMM: outer mitochondrial membrane; IMS: inter-membrane space; OXPHOS: oxidative phosphorylation complexes; schematic drawing after PubMed-ID 34502411 with modifications. Genetic and environmental causes as compiled in [109,110,111,112,113,114].

CLPP appears to be the most abundant family member in humans (mean abundance about 60 TPM). Its mutations cause the autosomal recessive Perrault syndrome type 3 (PRLTS3), but convey normal life expectancy [8,14,72,73,74,75,76,77,78,79]. PRLTS is a very rare phenotype, which is defined by primary ovarian insufficiency usually resulting in complete infertility, subsequent sensorineural hearing loss, and often, an age-associated leukodystrophy with prominent ataxia [16,80,81,82]. Data from one PRLTS3 male patient and CLPP-depleted mice show also complete male infertility with azoospermia to form part of the clinical picture [8,73,83]. A scheme summarizing the mitochondrial causes of similar infertility and deafness is shown in Figure 4. The mammalian findings clearly point to CLPP acting in the same pathway of mitochondrial transcription/translation as the many other known PRLTS disease proteins: mutations in PEO1/TWNK (encoding the nucleoid-associated helicase and primase Twinkle) cause PRLTS5 [73,75,84,85,86,87,88,89,90,91]. The homozygous variant Arg232Cys in TFAM (encoding the nucleoid-associated transcription factor A of mitochondria) was observed in one Pakistani PRLTS patient [77]. ERAL1 (encoding a mitochondrial rRNA chaperone involved in mitoribosome assembly) was associated with PRLTS6 in a Dutch family [40]. HARS2 (encoding the tRNA amino acid synthase for histidine) mutations in many patients and a mouse model trigger PRLTS2 [75,92,93,94,95,96,97]. LARS2 (encoding the tRNA amino acid synthase for leucine) is associated with PRLTS4 in many families [73,75,77,98,99,100,101,102,103,104,105]. RMND1 (encoding a mitoribosome-associated factor) mutations were found in a Portuguese proband and in two Polish sisters with Perrault-like syndrome plus renal involvement [106,107]. PRORP (encoding the metallonuclease subunit of mitochondrial RNAse P) caused Perrault syndrome in four families, sometimes resulting in developmental delay [108]. Thus, the phenotype produced by CLPP mutations with a combination of infertility and of sensorineural hearing impairment is highly specific. It corresponds to the typical consequences of mitochondrial DNA and RNA processing problems. Thus, mutations in the CLPP interactome lead to the combination of infertility with deafness, which is the hallmark of autosomal recessive PRLTS.

Meiotic arrest in testis with consequent azoospermia as upon CLPP depletion was observed in other mitochondriopathy mouse models (see Figure 4) only when mtDNA levels were manipulated [115,116]. Fertility reductions due to mitochondrial causes include large mtDNA deletions as in Kearns–Sayre syndrome (KSS), as well as mutations in the mtDNA polymerase POLG, the mtDNA replication helicase-primase Twinkle, thymidine kinase (TYMP), ribonucleotide reductase M2B (RRM2B), tRNA-LeuUUR, tRNA-His, tRNA-Ile, the tRNA amino acid synthases HARS2-LARS2-AARS2, CLPP, and the respiratory complex subunits ATPases 6/8 [111]. Thus, infertility such as primary ovarian insufficiency can be triggered by defects in mtDNA or mt-tRNA handling.

Sensorineural deafness upon mitochondrial dysfunction (see Figure 4) is mostly the consequence of mtDNA mutations 12183 G>A (encoding the tRNA for histidine, His), 3243 A>G (encoding the tRNA for leucine, Leu-UUR), and 7445 A>G (encoding the tRNA for serine, *Ser-UCN*) [117,118]. It is noteworthy that the 3243 A>5 mutation effects can be rescued by overexpression of the CLPX target candidate GFM2/EFG2 [119]. Mutations in both subsequent enzymatic steps, performed by the mitochondrial histidine tRNA synthase HARS2 and leucine tRNA synthase LARS2, are of course responsible for PRLTS2 and PRLTS4, respectively. Mitochondrial serine tRNA synthase SARS2 is under the control of a bidirectional promoter together with the mitoribosomal subunit MRPS12 at the chromosomal locus where autosomal dominant sensorineural deafness DFNA4 is inherited [120]. Furthermore, KARS mutants cause deafness via deficient *aminoacylation of mitochondrial lysine tRNA* [121]. It is interesting to note the adjacent positions on the heavy strand of the mitochondrial genome for tRNA-His (bp 12136–12206), tRNA-SerAGY (bp 12207–12265), and tRNA-LeuCUN (12207–12265). These clinical genetic findings implicate the tRNAs’ selectively for the amino acids His, Leu, and Ser in the pathogenesis of deafness, together with a specific region on the mtDNA H strand. Thus, the mitochondrial causes of deafness are strongly enriched for mtRNA-processing pathway components. The main subcellular site of this pathology may be the mitoribosome, where toxins such as aminoglycoside antibiotics were also documented to trigger deafness [122].

Very different clinical pictures result from mutations in non-RNP mitochondrial factors. The above clinical presentations do not noticeably impair the extracellular deposits of, e.g., collagen and are therefore markedly different from the congenital signs characteristic for cerebral, ocular, dental, auricular, and skeletal anomalies syndrome (CODAS). CODAS is caused by mutations in the Lon ortholog in humans, LONP1, as the general protease of the mitochondrial matrix [123,124]. The diagnostic hallmarks include craniofacial dysmorphia, cataracts, ptosis, median nasal groove, delayed tooth eruption, delayed epiphyseal ossification, metaphyseal hip dysplasia, vertebral coronal clefts, short stature, psychomotor developmental delay, and hearing loss [123,125].

## 7. Discussion

In contrast to a recent review that failed to distinguish the technical approaches and summarized that CLPP degradation substrates appear not conserved across phylogenesis [13], we attempted a careful evaluation of different approaches and organisms, together with the unbiased analysis of protein interaction knowledge and of complexomics data.

When the trap assay results are analyzed for consistency across phylogenesis in Table 1, it is important to note that 21 out of 48 putative ClpP substrates interact with single-strand nucleotide chains, so ribonucleoproteins are clearly overrepresented. Furthermore, practically all substrates associate either with a nucleotide cofactor, or with a nucleotide substrate, or both. Two substrate candidates (ACO2, LipA/LIAS) associate with FeS-clusters that are known to be very vulnerable to oxidative stress, and one substrate candidate (KatE) associates with heme.

Regarding the BioID protein–protein interaction evidence, CLPB was mainly found for complexes that generate and modify the mitochondrial polycistronic RNA and its derivatives until mRNA, tRNA, and mitoribosomes interact to achieve efficient translation initiation. The prominent interaction of CLPB with strong conservation across species is with the translation initiation factor MTIF2. The more abundant CLPX associated with more factors at the same complexes (e.g., with LRPPRC and again the IF-2 ortholog MTIF2). In addition, ClpX seems to serve functions at the nucleoid (TFAM and SSBP1), as well as translation elongation and termination. It associates preferentially with GTP-binding components of the protein synthesis machinery (such as GFM1, TUFM, MTG2), but also with the codon-independent translation release factor and stalled peptidyl-tRNA hydrolase ICT1. In comparison, CLPP was found at the same ribonucleoprotein complexes as CLPB and CLPX. In addition, CLPP more than CLPX interacted with factors of translation quality control, presumably before it acts as a degradation machine.

Overall, the sophisticated and valuable complexomics data on endogenous protein–protein interactions documented ClpB in *E. coli* bacteria to associate with MTIF2/HSPA9 orthologs that modulate translation initiation. In mouse heart, they suggested CLPB to act with TSFM/MRPL11 on translation elongation, with AUH on RNA degradation, and with HINT2 on nucleotide hydrolysis. In comparison, the mouse complexome profiles supported CLPX to act in association with GRSF1/TBRG4/LRPPRC on mtRNA processing and with GFM1/GFM2 on translation elongation.

The phenotypes of almost all mitochondriopathies discussed above show some degree of neurodegeneration due to demyelination, diagnosed as leukodystrophy upon brain imaging and as ataxia upon clinical-neurological evaluation. Dysfunction of the three AAA+ unfoldases CLPX, CLPB, and LONP1 causes distinctive features in peripheral tissues such as deficient heme biosynthesis, neutropenia, and collagen networks, respectively. Importantly, only CLPP mutations are responsible for the autosomal recessive combination of ovarian failure with sensorineural deafness, whose underlying mitochondrial causes usually affect the mtDNA/mtRNA processing protein complexes.

Overall, our analyses indicate a strong enrichment of ribonucleoprotein complexes among the CLPP targets. This concept is supported by recent findings in mouse mutants and patients that CLPP is crucial for the homeostasis of mitoribosomes and mitochondrial RNA granules [15,39], while LONP1 acts as a key determinant for the turnover, e.g., of the respiratory complex-I membrane arm [5].

**Figure 5:**
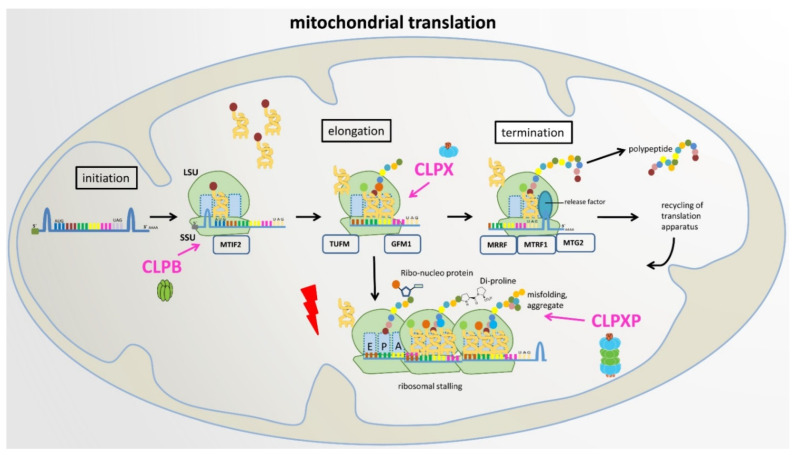
**Proposed roles of CLPB, CLPX, and CLPXP in mitochondrial translation**. LSU: large ribosomal subunit, SSU: small ribosomal subunit, E: exit site, P: peptide site, A: acceptor site in ribosomal machinery.

In view of the meta-analyses above, plausible roles of the mitochondrial peptidase components CLPB, CLPX, and CLPXP are schematically illustrated in Figure 5 below.

For substrate selection, CLPP depends on two AAA+ unfoldases, either the less-abundant and more rarely interacting ClpB or the abundant and more stably associated CLPX. In the context of CLPB, selectively, the translation initiation factor IF-2/MTIF2 was identified as a putative CLPP substrate/interactor repeatedly from bacteria to mitochondria. In the context of CLPX, the most frequently observed interactor was the translation elongation factor FusA/GFM1 from bacteria to mitochondria (Figure 2). It is important to note the observation that in ΔClpP *S. aureus* bacteria, accumulation of the translation elongation factor FusA (GFM1 ortholog) occurred selectively in the growth phase (and was similarly observed in growing ΔClpX *S. aureus* bacteria), while growth-arrested cells exhibited selective accumulation of the translation elongation factor TuF (TUFM) with the disaggregase ClpB. In contrast, the elongation factor TsF (TSFM) remained always stable [37]. Thus, the unfoldase activity of CLPB and CLPX seem to influence prominently the translation initiation and elongation steps, e.g., in mitochondria. In this process, MTIF2 protects formylmethionyl-tRNA and promotes its binding to the 30S mitoribosomal subunit, and TSFM then hydrolyzes GTP to trigger TUFM-dependent binding of any aminoacyl-tRNA to the A-site, before GFM1-dependent translocation to the P-site occurs [126]. If CLPB- or CLPX-mediated unfolding cannot repair the inappropriate conformation of any nascent incomplete RNA–protein complex or of any damaged RNP, the CLPXP degradation machine would assemble to linearize and degrade the RNP substrate. This concept provides a tentative explanation of why the dysfunction of ClpP causes the same PRLTS phenotype as mutations in the mitochondrial amino acid tRNA synthases HARS2 and LARS2. However, in this scenario, the translation factors such as MTIF2-TUFM-GFM1 and the unfoldases CLPX-CLPB would co-accumulate in CLPP-null organisms together with incomplete protein biosynthesis complexes. They would accumulate as an indirect consequence of delayed turnover, usually not as direct substrates of CLPP-mediated degradation.

In CLPP-null mouse heart complexomics, the disperse co-migration of accumulated CLPX with its accumulated interactions partners such as GFM1, GFM2, POLDIP2, GRSF1, LRPPRC, and FASTKD4 is best explained by their association with partially insoluble, misfolded, incomplete translation products of varying sizes with trapped misprocessed mRNA/tRNA, which cannot be degraded intra-mitochondrially in an efficient manner due to the absence of CLPP. It has been shown for bacterial translation that the peptide bond formation is especially slow with proline and that two adjacent prolines can even cause ribosome stalling [127,128]. Therefore, it is interesting to note that human/mouse sequences of COX1 as a key element of the rate-limiting respiratory complex-IV contain 2 double-prolines and 1 triple-proline (UniProt U5YWV7/Q9MD68), perhaps explaining why complex-IV biogenesis facilitators such as TACO1-SURF1-COX15 are prominent among CLPB and CLPXP interactors and the pathway of Cytochrome-C oxidase deficiency disease is enriched among CLPP interactions (see Figure 1, Figure 2 and Figure 3). In *Escherichia coli* and some further eubacteria, stalled ribosomes are rescued by the tmRNA system, which adds an alanine-rich ssrA tag as the degron to the C-terminus of the incomplete protein, thus directing degradation by the AAA+ ClpXP protease [129,130]. In most other bacteria, archaea, and some eukaryotes, stalled ribosomes are rescued by the addition of pure (quite insoluble) poly-alanine tails as degron to incomplete proteins, again triggering ClpXP-dependent degradation [130]. At present, it is unclear what the degron for incomplete mitochondrial proteins looks like and how the addition of degrons to an incomplete protein in human/mouse organisms would be triggered. It is possible that the continuous monitoring of ribosomal collisions and dynamics is integrated with the scrutiny of mRNA/tRNA turnover [131], before degradation signals are generated. Again, the observed accumulations and disperse migration of GFM1-POLDIP2-GRSF1-LRPPRC-FASTKD4 would then be secondary co-accumulations due to translation stalling and slowed turnover. In this context, it is noteworthy that ClpX was observed in *Mycobacterium tuberculosis* to interact with single-stranded DNA-binding protein (SSB). Thus, not only RNA processing such as transcription, hairpin folding, methylation/capping, cleavage, and translation, but also single-strand DNA processes appear to be modulated by the Clp AAA+ family members [132]. This concept is in good agreement with the main CLPP-null phenotypes, namely deafness that is frequently triggered by improper mtRNA processing and infertility with meiotic arrest, which was repeatedly described as a consequence of altered mtDNA processing. When CLPP is absent and such mtRNA/ss-mtDNA/protein complexes have to be degraded extra-mitochondrially, their relative hypomethylation would be expected to trigger cytosolic sensors that guard against bacterial/viral nucleic acid chains. This activation of innate immunity has indeed been observed in CLPP-null mouse tissues [133,134].

An important argument can immediately be raised against these notions: if RNA/ssDNA-protein complexes are the primary target of CLPB and CLPXP, why was the Clp AAA+ family named for its distinctive caseinolytic activity, since caseins are not known to associate with RNA or ssDNA? We suggest that both casein and misfolded RNP complexes are amphiphilic due to common features: Firstly, the casein family members contain a high number of proline amino acids, resulting in little tertiary structure. Thus, they assemble into hetero-polymeric complexes that are quite hydrophobic, forming micelles and undergoing liquid–liquid phase separation. Similarly, stalled mitoribosomal translation products would be proline-rich, unstructured, or misfolded with strong hydrophobicity, and RNP complexes are known to undergo phase separation upon misfolding due to cell stress [135]. Secondly, the casein micelle core consists of hydrophobic monomers that are connected by abundant calcium phosphate, while its surface contains relatively hydrophilic kappa-casein with many phosphorylated residues, resulting in an electric bilayer with negative charges outside [136,137]. Similarly, the outside backbone of RNA hairpin and cloverleaf structures are composed of hydrophilic phosphates, and calcium bursts in periods of cell stress trigger the formation of insoluble RNP aggregates [138]. In this context, it is relevant to note that substrate phosphorylation was reported to inhibit activation of ClpXP [139].

Another important doubt should be considered: although RNPs are enriched among the Clp AAA+ family interactions, this is not exclusive. There are many other matrix proteins that do not associate with single-stranded nucleotide chains, but do accumulate in ClpP-deficient organisms, and it remains unclear what their distinctive characteristics in various organisms are. We suggest that matrix proteins that associate with a nucleotide substrate such as ATP/GTP or with a nucleotide-like coenzyme such as pyridoxal-5′-phosphate/FAD/NAD+/NADPH may undergo similar misfolding problems in periods of cell stress and then also be targeted preferentially by the CLPXP degradation machine. Prominent examples in CLPP-deficient organisms include the highly conserved accumulation of the translation GTPases MTIF2/TUFM/GFM1/MTG2, the GTPase and mitochondrial dynamics regulator OPA1, the GTP-binding cell division regulator FtsZ, the GTP-sensing growth regulator CodY, the pyridoxal-5′-phosphate (P5P)-associated enzyme 5′-aminolevulinate synthase (ALAS), the P5P-associated enzyme ornithine aminotransferase (OAT), the P5P-associated enzyme serine hydroxymethyltransferase (SHTM2), the P5P-associated enzyme nitrogen-fixing bacteria S-like protein (NFS1), the FAD-associated enzymes such as ACAD9 or ACADSB, and the NAD/NADH-accepting malate dehydrogenase (MDH2). A prominent example of CLPX action on nucleotide-like cofactors may be P5P-associated ALAS: unfolding of ALAS by CLPX was shown to gate the binding of cofactor P5P, thus controlling the activity of this enzyme, in a function that is completely independent of CLPP [65,140]. In a similar manner, the principal role of CLPB and CLPX could be the disassembly of multimeric/aggregated proteins with nucleotides buried inside. In this concept, CLPP would be recruited only when misassemblies cannot be repaired, a problem that arises frequently during cell damage, e.g., with oxidative stress, or during the biosynthesis of polypeptides with double-prolines or triple-prolines such as the COX1 protein.

## 8. Proposal of a Plausible and Testable Scenario

Overall, the observations above indicate that most protein accumulations in CLPP-deficient organisms are indirect secondary effects due to slowed turnover or compensatory transcriptional inductions, so global proteome profiling in many mammalian cells/tissues has been of limited value to identify CLPP degradation substrates. In addition to documented trapping assays mainly in bacteria, it is useful to consider preliminary complexomics data from CLPP-null mice and protein interaction surveys from human cells.

Based on all such findings, we propose that the Clp AAA+ family associates preferentially with matrix proteins that show insoluble domains due to misfolding (e.g., after cell stress or after translation stalling by di-prolines), have phosphate groups masked or lost, and nucleotides or single-strand nucleotide chains stuck in their damaged conformation. Among the compensatory efforts of affected cells, CLPB would be responsible preferentially for translation initiation complexes, while CLPX would be responsible preferentially for translation elongation and recycling complexes. In cases where refolding is not successful, CLPP would bind to the unfoldases and perform its degradation activity. Upon failure of proteolysis, toxic aggregates can be extruded across the membrane, so mitochondrial affection can be rescued in the cytosol or membranes of eukaryotic organisms. Despite protective efforts in eukaryotes, eventually, the toxic accumulation of mtDNA and mtRNA will cause infertility, deafness, and broader neurodegeneration, as well as innate immunity activation during the ageing process in CLPP-mutant eukaryotes.

To assess whether this plausible scenario describes relevant physiological and stress-response mechanisms, a purification and reconstitution of CLPB and CLPX with CLPP into assembled complexes could be performed, and a quantification of proteolytic degradation rates of putative substrates in absence versus presence of RNA/ssDNA/nucleotide-cofactors could be documented. While our team has contributed via the generation and characterization of CLPP-null mice and the analysis of CLPP-mutant patient cells, such efforts are well beyond our expertise and should be carried out by a specialized mitochondrial biochemistry team. The insights gained would help to optimize the antibacterial usefulness of CLPP-targeting drugs and to advance the preventive therapies against several human diseases.

## Figures and Tables

**Table 1:** Summary of putative CLPP degradation substrates consistently defined by trap assay approaches and possibly supported in global proteome profiles. CLPX and GFM1 stand out. Relevant references are provided as PubMed-ID numbers. Grey background highlights RNA/ssDNA-interacting proteins.

	Trap assays					Proteome profiles					
	*E. coli*	*C. crescentus*	*S. aureus*	*P. anserina*	*H. sapiens* AML	*B. subtilis*	*S. aureus*	*A. thaliana*	*M. musculus* heart	*H. sapiens* fibroblasts	
PubMed-Ids	12667450, 16630889	23647068	12791139	26679294	26058080	15317791, 17981983	25743475	18754756	27797820, 32467259	34943861	
											nucleotide cofactors/substrates?
ACADSB				**+**	**+**				**+**		FAD
ACO2				**+**	**+**						[4Fe-4S] cluster
AhpC/At3g11630/PRDX			**+**	**+**				**+**			NADH
ClpB			**+**				**+**				ATP
ClpX	**+**	**+**	**+**	**+**	**+**	**+**				**+**	ATP
CodY			**+**				**+**				GTP
CS				**+**	**+**						-
DeaD/At5g26742	**+**							**+**			ATP
DnaJ/DNAJA3		**+**	**+**								ATP
DnaK/HSPA9	**+**		**+**	**+**				**+**	**+**		ATP
FabG/HSD17B4/8	**+**			**+**	**+**		**+**				NAD+
FtsA		**+**	**+**								ATP
FtsZ	**+**	**+**	**+**								GTP
FusA/GFM1	**+**			**+**			**+**	**+**	**+**	**+**	GTP, RNA-binding
GatA	**+**		**+**								-
GatB			**+**			**+**					-
GroEL/At2g28000/HSPD1	**+**		**+**					**+**			ATP
GyrB	**+**	**+**	**+**								ATP
InfB/MTIF2	**+**	**+**									GTP, RNA-binding
KatE	**+**					**+**					heme
LipA/LIAS	**+**		**+**								[4Fe-4S] cluster, SAM
McsB			**+**			**+**					ATP
MecA			**+**			**+**					-
MetK	**+**		**+**								SAM
MurA	**+**					**+**					UDP
MurC		**+**	**+**								UDP, NADP+
NrdA/E/H	**+**	**+**	**+**								ATP, ADP, CDP, UDP, GDP, dGTP, dTTP
OAT				**+**					**+**		P5P
OmpA	**+**	**+**									-
PdhC/DLAT/DLST			**+**	**+**		**+**					FAD, AcCoA, lipoate
PdhD/DLD			**+**	**+**		**+**					NAD+, FAD
Pnp/PNPT1	**+**		**+**						**+**		ATP, (nucleoside 5′-monophosphate)n
PpiB/At3g62030						**+**		**+**			-
RecA/DMC1		**+**	**+**			**+**					ATP, ssDNA-binding
RfaG/GspA		**+**				**+**					nucleotide-binding
Rho	**+**		**+**								ATP, RNA-binding
RplN	**+**		**+**								16S rRNA-binding
RplS	**+**		**+**								16S rRNA-binding
RplU	**+**		**+**								rRNA-binding
RpoS/SigB	**+**					**+**					DNA/RNA-binding
SdhB/SDHA/C		**+**			**+**						FAD, quinone, FeS-cluster
SecA	**+**		**+**				**+**	**+**			ATP, RNP-complex-binding
SHMT2				**+**	**+**						THF, P5P
Tkt			**+**			**+**					TPP
Tpx	**+**					**+**					-
Tsf/TSFM			**+**								GTP, RNA-binding
TufB/A/TUFM	**+**					**+**	**+**	**+**			GTP, RNA-binding
UvrA	**+**		**+**								ATP, ssDNA-binding

## Data Availability

This review does not report new data; instead, it provides meta-analyses of already published data.

## Abbreviations

16S	the RNA component in the 30S subunit of prokaryotic ribosomes
30S	prokaryotic small ribosomal subunit
2D-DIGE	two-dimensional difference gel electrophoresis
4Fe-4S	iron-sulfur cluster with [4Fe–4S] stoichiometry
Δ*clpP*	organism that lacks ClpP
Δ*clpX*	organism that lacks ClpX
*A. thaliana*	*Arabidopsis thaliana* (thale cress) is a small flowering plant
AAA+	protein superfamily of ring-shaped P-loop NTPases
AARS2	protein, alanyl—tRNA synthetase, mitochondrial
ACAD9	protein, acyl-CoA dehydrogenase family member 9 mitochondrial
ACADSB	protein, short/branched chain specific acyl-CoA dehydrogenase
AcCoA	metabolite, acetyl-coenzyme-A
ACO2	protein, aconitase 2, mitochondrial
ACSS1	protein, acyl-CoA synthetase short chain family member 1
ADP	nucleotide, adenosine di-phosphate
ALAS	protein, aminolevulinic acid synthase
ALAS1	protein, Delta-aminolevulinate synthase 1
ALDH6A1	protein, aldehyde dehydrogenase 6 family, member A1
AML	acute myeloid leukemia
APEX	ascorbic acid peroxidase
ATP	nucleotide, adenosine tri-phosphate
ATPase	enzymes that catalyze the decomposition of ATP into ADP
AUH	protein, AU RNA binding methylglutaconyl-CoA hydratase
*B. subtilis*	*Bacillus subtilis* is a Gram-positive, catalase-positive bacterium
BioID	biotinylation identification, a method to detect protein interactions
BN	blue native
C12orf65	chromosome 12 open reading frame 65 = MTRFR
C1QBP	complement C1q binding protein
*C. crescentus*	*Caulobacter crescentus* is a Gram-negative, oligotrophic bacterium
CARS2	protein, cysteinyl-tRNA synthetase 2, mitochondrial
CBR4	protein, carbonyl reductase 4
CCDC109B	mitochondrial calcium uniporter dominant negative beta subunit
CCDC90B	coiled-coil domain-containing protein 90B, mitochondrial
CDP	nucleotide, cytidine di-phosphate
CHCHD1	coiled-coil-helix-coiled-coil-helix domain containing 1 protein
ClpA	caseinolytic mitochondrial matrix peptidase chaperone subunit A
CLPB	caseinolytic mitochondrial matrix peptidase chaperone subunit B
CLPP	caseinolytic mitochondrial matrix peptidase chaperone subunit P
CLPX	caseinolytic mitochondrial matrix peptidase chaperone subunit X
CNE	clear native
CODAS	syndrome, cerebral, ocular, dental, auricular, skeletal anomalies
COX1	protein, mitochondrially encoded cytochrome C oxidase I
COX15	COX15 (yeast) homolog, cytochrome C oxidase assembly protein
COX5A	cytochrome C oxidase subunit 5A, mitochondrial
COX5B	cytochrome C oxidase subunit 5B, mitochondrial
COX6A1	cytochrome C oxidase subunit 6A1
COXPD1	combined oxidative phosphorylation defect type 1
COXPD3	combined oxidative phosphorylation defect type 3
COXPD39	combined oxidative phosphorylation defect type 39
COXPD4	combined oxidative phosphorylation defect type 4
CP29	chloroplast RNA-binding protein 29, in *A. thaliana*
CS	protein, citrate synthase, mitochondrial
DEAD-box	proteins with a DEAD (Asp-Glu-Ala-Asp) motif are RNA helicases
DFNA4	deafness, autosomal dominant, type 4
DFNA67	autosomal dominant nonsyndromic deafness 67
dGTP	nucleotide precursor, deoxy-guanosine triphosphate
DHX30	protein, ATP-dependent DExH-box helicase 30
DNA	deoxyribo-nucleic acid, polymer of polynucleotide chains
DNM1L	dynamin-1-like protein, a GTPase
dTTP	nucleotide precursor, deoxy-thymidine triphosphate
*E. coli*	*Escherichia coli*, a Gram-negative, facultative anaerobic bacterium
EFG2	elongation factor G 2, mitochondrial = GFM2
ERAL1	protein, era-like 12S mitochondrial rRNA chaperone 1
EXD2	protein, exonuclease 3′-5′ domain containing 2
FAD	coenzyme, flavin adenine dinucleotide
FASTKD2	protein, FAST kinase domain-containing protein 2, mitochondrial
FASTKD3	protein, FAST kinase domain-containing protein 3, mitochondrial
FASTKD4	protein, FAST kinase domain-containing protein 4, mitochondrial
FeS	iron-sulfur cluster
GATB	protein, glutamyl-tRNA amidotransferase subunit B
GDP	nucleotide, guanosine diphosphate
GFM1	protein, mitochondrial elongation factor G1
GFM2	protein, mitochondrial elongation factor G2
GO-term	Gene Ontology term
GRSF1	G-rich RNA sequence binding factor 1
GTP	nucleotide, guanosine-5′-triphosphate
GWAS	genome-wide association study
*H. sapiens*	*Homo sapiens* (human), the most abundant primate species
HARS2	protein, histidyl-tRNA synthetase 2, mitochondrial
HINT2	histidine triad nucleotide-binding protein 2, mitochondrial
HNRNPA2B1	heterogeneous nuclear ribonucleoprotein A2/B1
Hsp100	heat shock proteins with size around 100 kDa
HSPA9	heat shock protein family A (Hsp70) member 9
IARS2	isoleucyl-tRNA synthetase 2, mitochondrial
ICT1	peptidyl-tRNA hydrolase ICT1, mitochondrial = MRPL58
IDH3B	isocitrate dehydrogenase (NAD(+)) 3 non-catalytic subunit beta
IMM	inner mitochondrial membrane
IMS	inter-membrane space, mitochondrial
KARS	lysyl-tRNA synthetase 1
kDa	kiloDalton
KSS	Kearns–Sayre syndrome
LARS2	Leucyl-tRNA synthetase 2, mitochondrial
LONP1	mitochondrial Lon protease-like protein 1
LRPPRC	leucine-rich pentatricopeptide-repeat containing protein
*M. musculus*	*Mus musculus* (mouse) is a small mammal of the order Rodentia
MALDI-TOF	matrix-assisted laser-desorption-Ionization, with time-of-flight
MARCH5	membrane associated ring-CH-type finger 5 protein
MC4DN5	mitochondrial complex IV deficiency, nuclear type 5
MC4DN6	mitochondrial complex IV deficiency, nuclear type 6
MDH2	protein, malate dehydrogenase 2, mitochondrial
MEF	mouse embryonic fibroblasts
METTL15	12S rRNA M4C methyltransferase protein
METTL17	methyltransferase-like protein 17, mitochondrial
mRNA	messenger RNA
MRPP1	mitochondrial ribonuclease P protein 1 = tRNA (adenine-guanine(9)-N(1)) methyltransferase TRMT10C
MRPS12	mitochondrial small ribosomal subunit protein US12m
MRPS22	mitochondrial small ribosomal subunit protein MS22
MRPS26	mitochondrial small ribosomal subunit protein MS26
MRPS7	mitochondrial small ribosomal subunit protein US7m
MRPL11	mitochondrial large ribosomal subunit protein UL11m
MRPL13	mitochondrial large ribosomal subunit protein UL13m
MRPL18	mitochondrial large ribosomal subunit protein UL18m
MRRF	ribosome-recycling factor, mitochondrial
mtDNA	mitochondrial DNA
MTERF3	mitochondrial transcription termination factor 3
MTFMT	mitochondrial methionyl-tRNA formyl-transferase protein
MTG1	mitochondrial ribosome associated GTPase 1
MTG2	mitochondrial ribosome associated GTPase 2
mt-HSP70	mitochondrial heat shock protein 70 kDa = HSPA9
MTIF2	mitochondrial translational initiation factor 2
MTIF3	mitochondrial translational initiation factor 3
mt-lncRNA	mitochondrial long non-coding RNA
mt-mRNA	mitochondrial messenger RNA
mt-rRNA	mitochondrial ribosomal RNA
mt-tRNA	mitochondrial transfer RNA
MTRF1	mitochondrial translation release factor 1
MTRF1L	mitochondrial translation release factor 1 like
MTRFR	mitochondrial translation release factor in rescue
mtRNA	mitochondrial RNA
MTX2	mitochondrial outer membrane import complex protein 2
MUT	methylmalonyl-CoA mutase protein, mitochondrial
NAD+	nicotinamide adenine dinucleotide, oxidized
NADH	nicotinamide adenine dinucleotide, reduced
NADP+	nicotinamide adenine dinucleotide phosphate, oxidized
NADPH	nicotinamide adenine dinucleotide phosphate, reduced
NARS2	asparaginyl-tRNA synthetase 2, mitochondrial
NDUFA4	cytochrome C oxidase subunit FA4
NDUFAF1	NADH:ubiquinone oxidoreductase complex assembly factor 1
NEDMIAL	Neurodevelopmental disorder with severe motor impairment and absent language
NFS1	nitrogen-fixing bacteria S-like protein
NGRN	neugrin, neurite outgrowth associated
OAT	ornithine Aminotransferase
OMM	outer mitochondrial membrane
OPA1	optic atrophy 1 protein, mitochondrial dynamin-like GTPase
OTC	ornithine transcarbamylase protein
OXPHOS	oxidative phosphorylation (respiratory) chain complexes
*P. anserina*	*Podospora anserine*, a filamentous ascomycete fungus
P5P	cofactor, pyridoxal-5′-phosphate
PEO1	progressive external ophthalmoplegia 1 protein = Twinkle
PET112	glutamyl-tRNA amidotransferase subunit B = GATB
PMID	Public Library of Medicine (PubMed) identification number
PMPCA	peptidase, mitochondrial processing subunit alpha
PMPCB	peptidase, mitochondrial processing subunit beta
POLDIP2	DNA polymerase delta interacting protein 2
POLG	polymerase gamma catalytic subunit, mitochondrial
POLG2	mitochondrial DNA polymerase, accessory subunit
PPI	peptidyl-prolyl-cis-trans-isomerase, rotates proline peptide bond
PPI (in STRING)	Protein–protein-interaction
PRLTS	Perrault syndrome (autosomal recessive ovarian failure + deafness)
PRLTS2	Perrault syndrome type 2
PRLTS3	Perrault syndrome type 3
PRLTS4	Perrault syndrome type 4
PRLTS5	Perrault syndrome type 5
PRLTS6	Perrault syndrome type6
QRSL1	glutaminyl-tRNA amidotransferase subunit QRSL1
RMND1	protein, required for meiotic nuclear division 1 homolog
RNA	ribonucleic acid
RNAse P	RNA cleaving enzyme, for mitochondrial tRNA 5′-end processing
RNP	ribonucleoprotein
RPS12	small ribosomal subunit protein ES12
RPSA	small ribosomal subunit protein US2 = 67 KDa laminin receptor
RPUSD3	mitochondrial mRNA pseudouridine synthase D3
RPUSD4	mitochondrial RNA pseudouridine Synthase D4
RRM2B	ribonucleotide reductase regulatory TP53 inducible subunit M2B
rRNA	ribosomal RNA
*S. aureus*	*Staphylococcus aureus,* Gram-positive aerobic/anaerobic bacterium
SAM	*S*-adenosyl methionine, cosubstrate for methyl-group transfers
SARS2	protein, seryl-tRNA synthetase 2, mitochondrial
SDHA	succinate dehydrogenase complex flavoprotein subunit A
SDHC	succinate dehydrogenase complex subunit C
Ser-UCN	serine encoded by UCU, UCC, UCA, UCG (UCN) triplet
SHTM2	protein, serine hydroxymethyltransferase 2
SLC25A46	OMM protein, solute carrier family 25, member 46
SLC25A51	mitochondrial NAD+ transporter, solute carrier family 25 member 51
SLIRP	SRA stem-loop-interacting RNA-binding protein, mitochondrial
SSBP1	single-stranded DNA binding protein 1
ssDNA	single-stranded DNA
STRING	search tool for the retrieval of interacting genes/proteins
SURF1	Surfeit locus protein 1, cytochrome C oxidase assembly factor
TACO1	translational activator of cytochrome C oxidase I
TBRG4	transforming growth factor beta regulator 4 = FASTKD4
TCA	tricarboxylic acid cycle
TEFM	transcription elongation factor of mitochondria
TFAM	mitochondrial transcription factor A
THF	cofactor, tetrahydrofolate
TMEM70	transmembrane protein 70, mitochondrial; ATP synthase scaffold
tmRNA	transfer and messenger RNA, e.g., in *E. coli*
TPM	transcripts per million
TPP	thiamine pyrophosphate
tRNA	transfer RNA
tRNA-His	tRNA linked to histidine
tRNA-Ile	tRNA linked to isoleucine
tRNA-LeuCUN	tRNA linked to leucine, with codon CUU, CUC, CUA, CUG (CUN)
tRNA-LeuUUR	tRNA linked to leucine, with codon UUA, UUG (UUR)
tRNA-SerAGY	tRNA linked to serine, with codon AGU, AGC (AGY)
TRUB2	pseudouridine (Psi) synthase homolog 2, for COXI mt-mRNA
TSFM	Ts translation elongation factor, mitochondrial
TUFM	Tu translation elongation factor, mitochondrial
TWNK	Twinkle mtDNA helicase and primase = PEO1
TYMP	thymidine phosphorylase protein
UDP	nucleotide, uridine diphosphate
UQCRC1	ubiquinol-cytochrome C reductase core protein I
VWA8	von Willebrand factor A domain-containing protein 8
WARS2	tryptophan tRNA ligase 2, mitochondrial
YMR31	yeast mitochondrial ribosomal protein, =alpha-ketoglutarate dehydrogenase subunit
ZFE	Zentrale Forschungs-Einrichtung (Central research facility)
